# Rumen fermentation and epithelial gene expression responses to diet ingredients designed to differ in ruminally degradable protein and fiber supplies

**DOI:** 10.1038/s41598-022-06890-5

**Published:** 2022-02-21

**Authors:** C. B. Gleason, L. M. Beckett, R. R. White

**Affiliations:** 1 Department of Animal and Poultry Sciences, Virginia Tech, Blacksburg, VA 24061 USA; 2 Department of Animal Sciences, Purdue University, West Lafayette, IN 47907 USA

**Keywords:** Animal physiology, Fatty acids

## Abstract

Although numerous studies exist relating ruminal volatile fatty acid (VFA) concentrations to diet composition and animal performance, minimal information is available describing how VFA dynamics respond to diets within the context of the whole rumen environment. The objective of this study was to characterize how protein and fiber sources affect dry matter intake, rumen pH, fluid dynamics, fermentation parameters, and epithelial gene expression. Four diet treatments (soybean meal or heat-treated soybean meal and beet pulp or timothy hay) were delivered to 10 wethers. The soybean meals served as crude protein (CP) sources while the beet pulp and timothy hay represented neutral detergent fiber (NDF) sources. Feed intake, rumen pH, fluid pool size, and fluid passage rate were unaffected by treatment. Butyrate synthesis and absorption were greater on the beet pulp treatment whereas synthesis and absorption of other VFA remained unchanged. Both CP and NDF treatment effects were associated with numerous VFA interconversions. Expression levels of rumen epithelial genes were not altered by diet treatment. These results indicate that rumen VFA dynamics are altered by changes in dietary sources of nutrients but that intake, rumen environmental parameters, and the rumen epithelium may be less responsive to such changes.

## Introduction

Volatile fatty acids (VFA) produced by microbial fermentation of feed substrates in the rumen represent a key form of energy for the ruminant host animal as they supply approximately 70% of the metabolic energy that may be used for productive functions, such as growth and milk production^1^. The VFA profile produced in the rumen may exert a considerable degree of influence on the animal’s energy utilization for such activities because the VFA are utilized at differing efficiencies^2^. Improving our understanding of factors that determine fermentative outcomes would therefore help us to optimize the productive efficiency of ruminant livestock. Diet characteristics, such as forage-to-concentrate ratio, are known to influence VFA profiles and other variables that in turn may impact the VFA present in the rumen, including feed intake, rumen fluid dynamics, rumen pH, and rumen epithelial characteristics^3,4^. Studies examining diet effects on VFA have generally reported and discussed VFA responses to diet in terms of VFA molar proportions or concentrations. Concentrations, however, may be altered by fluid fluxes and should not be used for assessing or making predictions regarding dietary effects on fermentation parameters^5^. Research efforts should instead aim to characterize VFA responses to diet in terms of their production, interconversion, and absorption. These dynamic fluxes may be modeled using data obtained through stable isotope dilution experiments. To date, few published investigations have examined the effects of differing nutrient sources on VFA dynamics, rumen fluid dynamics, rumen pH, and expression of key epithelial genes concurrently. This lack of a holistic understanding of fermentation parameters represents a major gap in our knowledge of rumen function. There is also currently a considerable deficiency of data on fermentation dynamics of minor VFA. Although only present in the rumen in small quantities, the branched-chain minor VFA isobutyrate and isovalerate serve as essential substrate for certain rumen microbial species and are used in the synthesis of other branched-chain compounds such as amino acids and fatty acids^6,7^. The objective of this study was therefore to characterize how differing dietary sources of protein and fiber affect dry matter intake, rumen pH, fluid dynamics, fermentation dynamics of major and minor VFA, and epithelial gene expression using an ovine model (Table 1).

**Table 1:** Ingredients and nutrient inclusion for each treatment diet^a,b^.

	SBM-TH	HSBM-TH	SBM-BP	HSBM-BP
**Ingredient, % of DM**				
Alfalfa hay	10.0	9.9	10.1	10.1
Corn	16.0	15.7	19.2	19.9
Barley	16.0	15.7	19.2	19.9
Soybean meal	13.9	0.00	16.1	0.00
Soyplus	0.00	14.4	0.00	18.1
Timothy hay	23.5	20.6	0.00	0.00
Beet pulp	0.00	0.00	42.0	41.0
Wheat middlings	20.0	23.0	2.20	0.50
Trace mineral salt	0.74	0.88	0.54	0.55
Vitamin premix	0.74	0.88	0.54	0.55
**Nutrient, %**^c^				
DM	96.2	96.2	96.2	96.2
CP	17.0	16.8	16.6	16.1
NDF	29.8	30.7	29.6	31.0
ADF	13.5	12.9	13.7	14.2
Lignin	2.11	1.80	1.96	2.19
Starch	27.3	26.6	27.9	24.9
Ash	4.87	4.22	4.79	4.96

^*a*^*HSBM-TH* heat-treated soybean meal and timothy hay, *SBM-TH* soybean meal and timothy hay, *HSBM-BP* heat-treated soybean meal and beet pulp, *SBM-BP* heat-treated soybean meal and beet pulp.

^b^Alfalfa hay, corn, barley, wheat middlings, trace mineral salt, and vitamin premix were incorporated into pellets along with either soybean meal or Soyplus to create the SBM and HSBM treatments, respectively.

^c^Nutrient percentages are expressed on a DM basis except for DM, which is expressed on an AF basis.

## Results and discussion

### Dry matter intake, rumen pH, and rumen fluid parameters

Mean DMI for each treatment group are given in Table 2. Dry matter intake was unaffected by protein source (*P* = 0.59) and fiber source (*P* = 0.96), suggesting the diets had similar palatability despite observational differences in particle size across fiber sources caused by the inclusion of either pelleted beet pulp or long grass hay. No palatability issues were suspected as wethers were consuming appropriate amounts of feed for their body weights and typically left very little refusals. Diets were formulated to contain the same percentages of CP, NDF, and starch so the similar intakes may also indicate that the fermentation products of the diets elicited comparable satiety feedback responses in the brain. These results are consistent with those of Ghorbani and colleagues^8^, who observed similar intakes in sheep given different protein sources. Herrera-Saldana and Huber^9^ also reported no effect on intake in dairy cows when ingredients representing differing degradabilities of protein were fed. Beckett et al.^10^, however, reported decreased DMI in Holstein heifers on beet pulp compared to timothy hay and identified palatability as a likely cause of the intake difference. The experiment of Beckett et al.^10^ utilized a different form of timothy hay than we did (pelleted vs. long hay) and a different species, which may have contributed to the inconsistencies among observations.

**Table 2:** LS means for DMI and rumen variables as differentiated by diet along with *P* values for the effects of protein source, fiber source, and the interaction of protein and fiber source^a^.

Item	Diet				SEM	*P* value		
	SBM-TH	HSBM-TH	SBM-BP	HSBM-BP		Protein	Fiber	Protein × fiber
DMI, kg	1.56	1.48	1.52	1.51	0.12	0.59	0.96	0.57
Rumen pH	5.89	5.91	5.72	5.81	0.079	0.46	0.13	0.66
Rumen fluid volume, L	2.34	3.37	3.35	3.50	0.75	0.37	0.45	0.56
Rumen fluid Passage rate, %/h	8.63	6.47	3.90	3.81	2.7	0.60	0.16	0.69

^a^*HSBM-TH* heat-treated soybean meal and timothy hay, *SBM-TH* soybean meal and timothy hay, *HSBM-BP* heat-treated soybean meal and beet pulp, *SBM-BP* heat-treated soybean meal and beet pulp.

Similarly to DMI, rumen pH was also unaffected by protein source or fiber source (*P* = 0.46 and *P* = 0.13, respectively; Table 2). The physically effective NDF from forages and the lower digestibility of such NDF sources are known to help maintain rumen pH by stimulating the production of bicarbonate-containing saliva during chewing and by avoiding shifts towards more extreme rates of VFA production, which can occur when soluble carbohydrates are provided in abundance^11–13^. The neutral detergent soluble fiber (NDSF) fraction of beet pulp is predominantly pectin and, as a feedstuff, beet pulp contributes virtually no physically effective fiber when included in the diet^14^. Despite this, however, beet pulp has demonstrated the ability to stabilize rumen pH^15^. Pectin fermentation does not produce lactate and generally occurs to a lesser extent as pH decreases^14,16^. This is likely why we did not observe a significant difference in rumen pH values. Sheep are generally fed forage-heavy diets with little added concentrate, and normal rumen pH in sheep ranges from 6.4 to 6.8^17^. The diet treatments in this study were balanced to include 25% starch and, although mean rumen pHs were not low enough to be considered in a state of subacute ruminal acidosis (SARA), which is defined as below 5.5^17,18^, the low mean values indicate animals may have experienced SARA at some points. The diets used in this study were intended to be reflective of feedlot diets (with a fairly low forage-to-concentrate ratio) and SARA is not an uncommon issue in feedlot animals. However, none of our wethers exhibited signs of suffering from acidosis (anorexia, discomfort, diarrhea, etc.) during the course of the trial and there was no evidence of rumen epithelial damage during tissue sampling.

Rumen fluid volume and passage rate also demonstrated no response to protein source (*P* = 0.37 and *P* = 0.60, respectively; Table 2) or fiber source (*P* = 0.45 and *P* = 0.16, respectively; Table 2). Rumen fluid volume and passage rate can be influenced by factors such as osmolality (from fluid flux across the rumen wall) and water intake^19^. Although osmolality and water intake were not measured in our study, it appears that our diet treatments did not exert a noticeable influence on them. Links between water intake and DMI have been reported previously^20^. Differences in water intake were not expected and we did not observe differences in DMI among our treatments, which may indirectly help to explain why there were no differences in rumen fluid volumes or passage rates. Mean fluid passage rates for TH diets were consistent with those reported by other investigations in sheep consuming similar diets^21,22^. Mean values for the BP diets, however, appeared lower than in previous literature and the reason is not readily apparent. We still did not detect a statistically significant difference (*P* = 0.16) between treatment means. The fluid passage estimation technique using polyethylene glycol (PEG) was performed identically between all animals and experimental periods. Issues with inconsistent results and data variability using PEG to measure rumen fluid passage have been raised in the past^23^, and this could simply be a drawback of the method itself^24^. We chose to use PEG, however, as an alternative to other markers such as CoEDTA and CrEDTA, which have been demonstrated to dissociate in the rumen by Hall and Van Soest^25^. Mean rumen fluid volumes were fairly consistent with those reported by Ulyatt^26^, who also utilized PEG as a fluid marker in adult sheep as we did. Our values, however, appeared to be underestimations when compared to the observations of other researchers^27,28^. Inconsistent reports in literature of ovine rumen fluid volumes estimated using PEG have been discussed and were attributed mainly to variation between experimental conditions of different studies^28^. There is also evidence that sheep fed every 2 h (as in our study) have decreased rumen volumes compared to when they are fed every 12 h^29^. While the usage of PEG as a marker may not yield absolute rumen fluid volumes, it is considered an adequate technique for comparisons of the effects of different dietary treatments^26^. Bailing the rumen to obtain actual volumes would have been optimal, but we were unable to utilize this technique due to concerns of disrupting the rumen microbial populations (data presented in Gleason et al.^30^).

### Fermentation parameters

Concentrations of all VFA except isobutyrate were observed to increase in response to BP treatment compared to TH (*P* < 0.006; Table 3). No concentrations of VFA demonstrated a response to protein source (*P* > 0.10; Table 3). Although VFA concentrations may be used to describe effects of diet treatments under specific ruminal conditions, their sensitivity to fluid inflow and outflow make them inappropriate measurements for drawing general conclusions about dietary influences of fermentation or for attempting to make comparisons across different studies^5^. Concentrations of VFA were calculated for this investigation as an input for the VFA dynamics modeling process and their values are presented in this writing only for consistency with reporting practices in the literature. The remainder of this discussion will therefore focus on describing dietary impacts on ruminal VFA in terms of their molar proportions and fermentation dynamics rather than concentrations.

**Table 3:** LS means for ruminal VFA concentrations and molar proportions as differentiated by diet along with *P* values for the effects of protein source, fiber source, and the interaction of protein and fiber source^a^.

Item	Diet				SEM	*P* value^b^		
	SBM-TH	HSBM-TH	SBM-BP	HSBM-BP		Protein	Fiber	Protein × fiber
**Concentration, mM**								
Acetate	3.05	3.09	3.65	3.63	0.17	0.99	**< 0.001**	0.84
Propionate	1.11	0.992	1.41	1.23	0.098	0.11	**0.0053**	0.76
Butyrate	0.582	0.668	0.846	0.897	0.076	0.37	**0.0036**	0.82
Valerate	0.0830	0.0808	0.126	0.121	0.012	0.76	**0.0019**	0.92
Isovalerate	0.0476	0.0499	0.0927	0.0740	0.0082	0.27	**< 0.001**	0.17
Isobutyrate	0.0437	0.0461	0.0516	0.0438	0.0030	0.38	0.26	**0.081**
**Molar proportion, % mol**								
Acetate	63.1	62.7	60.5	61.6	1.3	0.77	**0.090**	0.47
Propionate	21.9	20.1	22.1	20.2	1.4	0.16	0.86	0.94
Butyrate	11.6	13.5	13.4	14.6	1.0	0.12	0.23	0.72
Valerate	1.65	1.65	1.96	1.94	0.15	0.97	**0.056**	0.91
Isovalerate	0.951	1.03	1.52	1.25	0.16	0.52	**0.0065**	0.24
Isobutyrate	0.873	0.964	0.852	0.781	0.067	0.80	**0.079**	0.16

^a^*HSBM-TH* heat-treated soybean meal and timothy hay, *SBM-TH* soybean meal and timothy hay, *HSBM-BP* heat-treated soybean meal and beet pulp, *SBM-BP* heat-treated soybean meal and beet pulp.

^b^Bolded values indicate significance or a tendency.

Volatile fatty acid molar proportions were minimally impacted by treatment. The only significant effect was observed for isovalerate molar proportions, which were greater on the BP treatment compared to TH (*P* = 0.0065; Table 3). The BP treatment also tended to increase molar proportions of valerate (*P* = 0.056) whereas TH was associated with trends for greater percentages of acetate and isobutyrate (*P* = 0.090 and *P* = 0.079, respectively). These results are mostly consistent with the observations of Beckett et al.^10^, who reported greater acetate proportion in response to TH, a tendency for isobutyrate proportion to increase on TH, and a tendency for valerate proportion to increase on BP. Interestingly, Beckett and colleagues^10^ observed isovalerate proportions to increase on TH instead of BP, in direct conflict with our observation. Even though isovalerate only represents a minor proportion of the rumen VFA, it is an important substrate used by rumen microbes to synthesize leucine and other branched-chain compounds^6^. The inconsistency of isovalerate response to these differing fiber treatments therefore merits further investigation. The overall lack of significance for the other VFA proportions, however, indicates that the rumen microbes are able to use different substrate sources to generate fairly similar fermentative profiles.

As discussed above, we observed some inconsistencies between our rumen fluid dynamics estimates and those of a few previous investigations utilizing the PEG marker technique. Such inconsistencies are not uncommon throughout the literature. We therefore suggest a cautious interpretation of the VFA absorption values themselves due to them being calculated using the fluid dynamics data. Because we cannot guarantee that our absorption estimates are equal to the actual absolute values, we instead will emphasize and discuss the presence or absence of treatment effects on what we will term the *apparent* absorption of VFA. Based on our estimations, butyrate synthesis rate and apparent absorption fluxes increased in response to treatment with BP compared to TH (*P* = 0.015 and *P* = 0.045, respectively; Table 4). Synthesis and apparent absorption of other VFA were not affected by diet treatment, nor was diet associated with altered fluid-mediated exit of any VFA (Table 4). This suggests that the changes in dietary sources of fiber and protein that we utilized in our experiment did not alter rumen function sufficiently to elicit shifts in the production, absorption, or outflow of most VFA. A reason for this lack of treatment effect on the VFA, excluding butyrate, was not readily apparent. Investigations examining dietary effects on VFA production and absorption rates are scarce, making extensive comparisons with our findings to the current available literature difficult and further underscoring the need for additional investigation. Esdale and colleagues^31^ reported production rate increases in both butyrate and propionate when corn silage was fed compared to alfalfa hay. Sutton et al.^32^ similarly observed considerable increases in the rate of propionate production as diet concentrate level was increased but, interestingly, reported a slight decrease in the rate of butyrate production. While the diet treatments utilized in these two investigations would be expected to contain differing concentrations of nutrients such as starch and protein (unlike our diets), they nevertheless indicate that rates of butyrate production are sensitive to diet characteristics. Work by Qumar et al.^33^ has demonstrated that absorption of VFA, including butyrate, can be altered by concentrate feeding strategy; however, their assessment compared continuous versus interrupted concentrate feeding schedules whereas our treatments were based on feed ingredients. In addition to pectin, the carbohydrate fraction of beet pulp is composed largely of hemicellulose^34^, which is a preferred fermentative substrate of the rumen bacterial species *Butyrivibrio fibrisolvens*^35^. As its name suggests, *B. fibrisolvens* is a prominent producer of butyrate^35^. It is therefore possible that the BP diet treatment stimulated an increase in butyrate synthesis through the provision of hemicellulose to a key butyrate-producing bacterial population. Dietary effects on the rumen microbiome are examined separately in Gleason et al.^30^ Of the ruminal VFA fermented, butyrate is the one primarily metabolized by the rumen epithelium as an energy source^36^. The increased apparent absorption of butyrate observed in response to the BP treatment suggests that the epithelium may be altered to increase its uptake of this VFA; however, as will be discussed below, no significant changes in rumen epithelial gene expression due to diet treatment (including any associated with VFA transport) were observed. Regardless of the exact microbial or epithelial mechanisms potentially in play, our results demonstrate that butyrate synthesis and absorption may be altered by differences in the dietary source of fiber provided to the animal. Further research is necessary to better elucidate the impact of dietary changes on synthesis and absorption of butyrate, in addition to the other VFA for which we did not observe treatment effects.

**Table 4:** LS means for VFA dynamics measurements as differentiated by diet along with *P* values for the effects of protein source, fiber source, and the interaction of protein and fiber source^a,b^.

Measurement	Diet				SEM	*P* value^c^		
	SBM-TH	HSBM-TH	SBM-BP	HSBM-BP		Protein	Fiber	Protein × fiber
**Synthesis, mmol/h**								
Acetate	22.3	15.7	17.8	28.4	6.9	0.73	0.50	0.18
Propionate	12.3	4.44	6.11	14.2	6.0	0.99	0.77	0.20
Butyrate	1.53	1.07	2.87	7.41	1.6	0.15	**0.015**	**0.083**
Valerate	1.82 × 10^−4^	1.09 × 10^−5^	4.10 × 10^−6^	6.33 × 10^−5^	9.63 × 10^−5^	0.57	0.53	0.26
Isovalerate	1.06 × 10^−5^	9.52 × 10^−5^	6.18 × 10^−4^	7.96 × 10^−4^	5.06 × 10^−4^	0.80	0.22	0.93
Isobutyrate	4.65 × 10^−5^	3.23 × 10^−8^	4.10 × 10^−9^	3.15 × 10^−7^	2.33 × 10^−5^	0.34	0.34	0.33
**Absorption rate, mmol/mmol/h**								
Acetate	0.755	0.591	0.716	0.699	0.090	0.34	0.70	0.69
Propionate	0.763	0.629	0.762	0.725	0.077	0.29	0.54	0.53
Butyrate	0.704	0.563	0.618	0.564	0.13	0.46	0.74	0.74
Valerate	0.741	0.578	0.745	0.644	0.11	0.26	0.76	0.78
Isovalerate	0.564	0.696	0.531	0.542	0.13	0.58	0.47	0.64
Isobutyrate	0.671	0.590	0.738	0.763	0.10	0.75	0.21	0.56
**Absorbed, mmol/h**								
Acetate	13.0	9.01	11.7	23.6	5.9	0.45	0.22	0.16
Propionate	9.33	4.27	6.47	12.6	4.1	0.90	0.52	0.20
Butyrate	3.11	3.65	4.27	7.45	1.3	0.12	**0.045**	0.25
Valerate	0.948	0.525	0.915	1.21	0.31	0.77	0.19	0.16
Isovalerate	0.229	0.506	0.564	0.643	0.20	0.34	0.22	0.59
Isobutyrate	0.387	0.324	0.429	0.785	0.21	0.39	0.16	0.23
**Passage with fluid, mmol/h**								
Acetate	4.31	2.24	1.14	1.53	2.2	0.69	0.36	0.56
Propionate	3.42	1.19	0.623	0.806	1.7	0.53	0.34	0.47
Butyrate	1.00	0.970	0.500	1.17	0.76	0.62	0.82	0.59
Valerate	0.338	0.180	0.0976	0.124	0.18	0.69	0.38	0.58
Isovalerate	0.112	0.103	0.0988	0.0800	0.083	0.84	0.80	0.94
Isobutyrate	0.117	0.0993	0.0401	0.0402	0.067	0.90	0.31	0.89

^a^*HSBM-TH* heat-treated soybean meal and timothy hay, *SBM-TH* soybean meal and timothy hay, *HSBM-BP* heat-treated soybean meal and beet pulp, *SBM-BP* heat-treated soybean meal and beet pulp.

^b^mmol/mmol/h = fraction of carbon per hour.

^c^Bolded values indicate significance or a tendency.

Contrasting with the lack of dietary effect on many of our other response variables of interest, numerous changes in VFA interconversion rates and fluxes were observed in response to both fiber and protein treatments (Table 5; nonsignificant interconversions are located in Supplementary Table S1). Treatment with BP increased or tended to increase carbon fluxes towards isovalerate from acetate (*P* = 0.0064), propionate (*P* = 0.0016), valerate (*P* = 0.0014), and isobutyrate (*P* = 0.091). The increased shift towards isovalerate helps to explain the greater molar proportions observed for this VFA on the BP treatment. Rates of interconversion from acetate and propionate to isovalerate tended to increase as well (*P* = 0.059 and *P* = 0.075, respectively). Beet pulp treatment was also associated with an increased flux from acetate to valerate (*P* = 0.0097) and a tendency for increased flux from isobutyrate to propionate (*P* = 0.075). Interconversion rates observed to increase or tending to increase when TH diets were fed were predominantly involving propionate. These exchanges were the fractional interconversion rate from acetate to propionate (*P* = 0.0031), propionate to butyrate (*P* = 0.088), and propionate to valerate (*P* = 0.0080). The flux from acetate to propionate was also increased (*P* = 0.015). Interconversion rates that increased or tended to increase in response to the HSBM treatment compared to the SBM treatment included propionate to valerate (*P* = 0.023), valerate to isovalerate (*P* = 0.096), and isobutyrate to butyrate (*P* = 0.047). The corresponding fluxes from valerate to isovalerate and isobutyrate to butyrate were greater as well (*P* = 0.030 and *P* = 0.035, respectively). The SBM treatment tended to increase the rate of transfer from butyrate to acetate and propionate (*P* = 0.061 and *P* = 0.055, respectively).

**Table 5:** LS means for significant and trending VFA interconversions as differentiated by diet along with *P* values for the effects of protein source, fiber source, and the interaction of protein and fiber source^a,b^.

Measurement	Diet				SEM	*P* value^c^		
	SBM-TH	HSBM-TH	SBM-BP	HSBM-BP		Protein	Fiber	Protein × fiber
**Interconversion rate, mmol/mmol/h**								
Acetate to propionate	0.355	0.404	0.176	0.0972	0.063	0.82	**0.0031**	0.32
Acetate to isovalerate	0.0376	0.0245	0.0624	0.0593	0.014	0.57	**0.059**	0.73
Propionate to butyrate	0.222	0.478	0.224	0.151	0.11	0.31	**0.088**	**0.084**
Propionate to valerate	0.0582	0.0960	0.0323	0.0502	0.017	**0.023**	**0.0080**	0.32
Propionate to isovalerate	0.0133	0.0166	0.0341	0.0433	0.013	0.61	**0.075**	0.81
Butyrate to acetate	0.446	0.262	0.317	0.184	0.12	**0.061**	0.19	0.74
Butyrate to propionate	0.407	0.244	0.319	0.123	0.092	**0.055**	0.22	0.83
Valerate to acetate	0.477	0.261	0.203	0.451	0.12	0.90	0.73	**0.077**
Valerate to isovalerate	0.0286	0.0811	0.0494	0.132	0.037	**0.096**	0.34	0.68
Isobutyrate to butyrate	0.170	0.477	0.332	0.535	0.12	**0.047**	0.36	0.66
**Interconversion flux, mmol/h**								
Acetate to propionate	6.26	7.28	1.91	1.62	1.8	0.84	**0.015**	0.72
Acetate to valerate	1.17	0.521	1.15	1.75	0.36	0.89	**0.0097**	**0.0090**
Acetate to isovalerate	0.456	0.379	1.20	1.77	0.47	0.38	**0.0064**	0.26
Propionate to isovalerate	0.142	0.119	0.421	0.734	0.22	0.32	**0.016**	0.26
Valerate to isovalerate	0.0288	0.0614	0.0777	0.219	0.040	**0.030**	**0.014**	0.14
Isobutyrate to propionate	0.0740	0.0713	0.189	0.223	0.087	0.81	**0.075**	0.77
Isobutyrate to butyrate	0.0920	0.403	0.211	0.495	0.14	**0.035**	0.40	0.91
Isobutyrate to isovalerate	0.0163	0.0232	0.0515	0.0987	0.040	0.36	**0.091**	0.49

^a^*HSBM-TH* heat-treated soybean meal and timothy hay, *SBM-TH* soybean meal and timothy hay, *HSBM-BP* heat-treated soybean meal and beet pulp, *SBM-BP* heat-treated soybean meal and beet pulp.

^b^mmol/mmol/h = fraction of carbon per hour.

^c^Bolded values indicate significance or a tendency.

Previous investigations have established that VFA interconversions are impacted to some extent by diet characteristics. Esdale et al.^31^ reported increased carbon transfer from acetate to butyrate associated with feeding corn silage compared to alfalfa hay. Minimal interconversion with propionate was observed on either treatment^31^. Work by Sutton et al.^32^, however, found that 13% and 10% of butyrate carbon originated from propionate at diet levels of 60% and 90% concentrate, respectively. An additional investigation utilizing high and low forage-to-concentrate ratios as diet treatments described fluxes from acetate to propionate and acetate to butyrate, with minimal conversion from butyrate back to acetate^37^. The explanatory power of these findings is limited by the fact that all of their diet treatments essentially only represent varying levels of forage-to-concentrate ratio. However, they still illustrate that differences in fermentative substrate type can influence fluxes between the three major VFA. To the best of our knowledge, no published studies in existence at the time of this writing have reported diet effects on interconversions involving the minor rumen VFA. Our results therefore appear to be among the first characterizations to be made available of diet-driven alterations in the interconversion dynamics between both major and minor VFA.

The precise manner in which the differences in dietary sources of fiber and protein are altering the exchanges between the various VFA is not readily apparent, but it is probable that diet is influencing ruminal variables not examined in this study that play a role in driving interconversions. As explained by Ungerfeld and Kohn^38^ using thermodynamics principles, rumen VFA interconversions are able to occur because the change in Gibbs free energy (ΔG) for the VFA interconversion reactions can be inferred to be near zero and, thus, near equilibrium based on the fact that the reactions producing acetate, propionate, and butyrate from pyruvate have similar ΔG values. The ability of VFA to interconvert is known to be an important tool utilized by rumen microbes to regenerate intermediates needed for particular metabolic processes^39^. A possible explanation for the diet-induced responses in interconversions we observed is that the microbes are adapting to changes in the ruminal availability of fermentative substrates provided by our particular treatments by adjusting aspects of their VFA interconversion activities. Additional investigation is required to determine if this is indeed the route by which diet is exerting influence on interconversion dynamics.

### Epithelial gene expression

Expression levels of the rumen epithelial genes under investigation were not significantly altered by treatment (*P* > 0.05; Table 6). However, expression of the genes AKT1 and NHE2 tended to increase (*P* = 0.088 and *P* = 0.075; respectively) in response to the BP treatment compared to TH. AKT1 codes for Serine-threonine protein kinase B, an enzyme involved in protein synthesis regulation^40^. Specific dietary influences of AKT1 gene expression in the rumen epithelium are largely unknown. Recent investigations into a possible link between AKT1 and feed intake have yielded conflicting results^41,42^. NHE2 is expressed within epithelial cells and is involved with the intracellular regulation of sodium levels and pH^43^. Gene expression of NHE2 was found to be upregulated in a caprine model when the diet concentrate fraction was increased^4^, indicating this gene may be regulated by diet characteristics. Interestingly, Beckett and colleagues^10^ observed that a small number of epithelial gene expression levels were affected or tended to be affected by beet pulp vs. timothy hay treatment but these did not include AKT1 or NHE2. We, conversely, did not observe any alteration in expression of the genes identified by Beckett et al.^10^ as sensitive to fiber source (NHE3 and HSP70) or as showing a trend toward sensitivity to fiber source (BDH1 and MCT4). Beckett and colleagues^10^ also reported a difference in rumen pH associated with the fiber treatment. As mentioned above, we observed no treatment effect on rumen pH. Other investigations into epithelial gene expression have noted that dietary alterations associated with changes in gene transcription may be exerting their effects via the creation of acidic conditions in the rumen^4,44,45^. Together, these observations and ours indicate that changes in the type of fermentative substrate (e.g. fiber vs. starch) that result in ruminal pH shifts are more likely than changes in the source of the same nutrient (e.g. forage fiber vs. non-forage fiber) to alter epithelial gene expression. Our finding of little to no dietary effect on gene expression is important because it suggests that varying nutrient sources do not necessarily result in altered epithelial function, which indicates a certain level of flexibility may be possible with the usage of particular diet components. It must be remembered, however, that while gene expression levels can indicate changes in the molecular response to a treatment, we cannot infer the level of functional protein from them. Current knowledge of rumen epithelial protein expression level responses to diet is limited, and additional research is therefore necessary to elucidate any impacts of nutrient source alterations on the actual abundances of key proteins involved in rumen epithelial function. Investigations by Hollmann et al.^44^ and Zhang et al.^45^ into the effects of high-concentrate diets on rumen epithelial protein expression provide useful experimental methods for continued research.

**Table 6:** LS means for rumen epithelial gene expression (2^−ΔCt^) as differentiated by diet along with *P* values for the effects of protein source, fiber source, and the interaction of protein and fiber source^a^.

Gene	Abbreviation	Diet				SEM	*P* value^b^		
		SBM-TH	HSBM-TH	SBM-BP	HSBM-BP		Protein	Fiber	Protein × fiber
Beta-hydroxybutyrate dehydrogenase	BDH1	0.636	7.93	3.82	20.3	8.9	0.19	0.19	0.64
Claudin-1	CLDN1	1.47	0.871	1.10	0.902	0.32	0.60	0.21	0.51
Gap junction protein alpha 1	GJA1	0.572	0.314	0.281	0.359	0.14	0.71	0.26	0.20
Heat shock protein 70	HSP70	0.0515	0.0637	0.275	0.143	0.071	0.44	0.61	0.44
3-Hydroxy-3-methylglutaryl-CoA synthase 2	HMGCS2	80.2	50.1	16.0	185	64	0.44	0.45	0.11
Monocarboxylate transporter isoform 1	MCT1	1.76	1.98	1.77	1.50	0.59	0.95	0.61	0.66
Monocarboxylate transporter isoform 2	MCT2	0.00552	0.0758	0.110	0.00291	0.059	0.95	0.60	**0.090**
Monocarboxylate transporter isoform 4	MCT4	0.0248	0.113	0.118	0.0264	0.072	0.83	0.86	0.20
Serine-threonine protein kinase B	AKT1	0.806	3.98	4.82	15.6	6.5	0.27	**0.088**	0.63
Sodium-hydrogen exchanger isoform 1	NHE1	0.0582	0.0921	0.0758	0.133	0.069	0.53	0.56	0.86
Sodium-hydrogen exchanger isoform 2	NHE2	1.15	1.89	1.82	4.06	0.98	0.18	**0.075**	0.42
Sodium-hydrogen exchanger isoform 3	NHE3	0.870	0.627	0.821	0.823	0.18	0.11	0.35	0.46

^a^*HSBM-TH* heat-treated soybean meal and timothy hay, *SBM-TH* soybean meal and timothy hay, *HSBM-BP* heat-treated soybean meal and beet pulp, *SBM-BP* heat-treated soybean meal and beet pulp.

^b^Bolded values indicate significance or a tendency.

### Conclusions

This work sought to characterize the responses of dry matter intake, rumen fluid parameters, rumen pH, VFA dynamics, and rumen epithelial gene expression levels to diets differing in sources of fiber and protein. Using an ovine model, differences in dietary sources of fiber and protein were found to elicit shifts in rumen VFA profiles and dynamics, especially in interconversions among VFA. Treatment differences, however, may have been insufficient to exert a significant degree of change on feed intake, rumen environmental characteristics, and gene expression levels in the rumen epithelium. The complex, interrelated process of converting feed to VFA is currently not well understood, and our investigation helps to bridge this knowledge gap by providing novel descriptions of carbon exchanges with minor VFA as impacted by diet characteristics. Future work should investigate the effects of these diet variables on postabsorptive metabolism of VFA and animal performance parameters.

## Methods

### Animals, experimental design, and treatments

All animal use and procedures in this study were approved by the Virginia Tech Institutional Animal Care and Use Committee (Protocol #18-096) and the study was carried out in accordance with the guidelines of Virginia Tech University and with ARRIVE guidelines. Ten ruminally cannulated commercial wethers (Suffolk, Dorset, or Suffolk × Dorset) were housed individually in covered pens at the Smithfield Farm, Virginia Tech, Blacksburg, VA. Wethers were approximately 1.5 years of age and an average of 62.1 ± 6.6 kg body weight (BW) at the start of the experiment. Wethers were blocked by initial BW and randomly assigned to treatments in a partially replicated 4 × 4 Latin square. Treatment assignments were arranged factorially (2 × 2) and included feedstuffs thought to supply differing rumen degradabilities of CP and NDF. Soybean meal (SBM) and heat-treated soybean meal (HSBM) represented the CP sources with high and low rumen degradabilities, respectively^46^. These meals were pelleted along with alfalfa, corn, barley, wheat middlings, trace mineral salt, and a sheep vitamin-mineral premix (Table 1). Pelleted beet pulp (BP) and long timothy hay (TH) were utilized as the NDF sources, with BP expected to undergo swifter fiber degradation compared to TH. Diets (Table 1) were prepared daily by combining the appropriate protein pellet with the appropriate fiber source to yield the 4 treatments: highly degradable CP plus lowly degradable NDF (SBM-TH), highly degradable CP plus highly degradable NDF (SBM-BP), lowly degradable CP plus lowly degradable NDF (HSBM-TH), and lowly degradable CP plus highly degradable NDF (HSBM-BP). Each animal consumed each of the 4 diets for a 21-d period. Animals were gradually adapted to their diets during the first 3 d then consumed 100% of the diet for the remaining 18 d. Diets were provided once daily at 0800 h from d 0–15. Animals were fed 1/12th of their daily ration every 2 h for the remainder of the period (d 16–20) in an effort to more closely mimic a metabolic steady state during the infusion process (detailed below). Refusals were collected daily throughout the experiment to calculate dry matter intake (DMI). Clean, fresh water was available at all times.

### Feed analysis

Feed samples were dried for 24 h at 55 °C in a forced-air oven (Thermo Scientific Heratherm Advanced Protocol Ovens Model 51028115, Fisher Scientific; Waltham, MA) and ground to pass through a 1 mm screen of a Wiley mill (Model 4, Thomas Scientific; Swedesboro, NJ). Dry matter percentage was determined by drying ground samples for 12 h at 100 °C. Ash content was obtained through combustion in a muffle furnace (Sybron Thermolyne FA1730, Fisher Scientific; Waltham, MA) for 12 h at 500 °C. Concentrations of NDF and acid detergent fiber (ADF) were determined using the Ankom200 Fiber Analyzer (Ankom Technology; Macedon, NY). Sodium sulfite (Sodium Sulfite, anhydrous, Fisher Scientific; Waltham, MA) and α-amylase from *Bacillus licheniformis* (Thermostable Amylase HTL, BIO-CAT; Troy, VA) were utilized in the NDF analysis^47^. Residues from ADF analysis were agitated for 3 h in 72% sulfuric acid in a 2 L beaker on a rocking platform (Flask Dancer, Boekel Scientific; Feasterville-Trevose, PA) to obtain acid detergent lignin concentrations. Nitrogen content was determined by combustion analysis using a Vario El Cube CN analyzer (Elementar Americas Inc.; Mount Laurel, NJ) and CP concentration was calculated as N percentage × 6.25. Starch concentration was determined following the acetate buffer method^48^ with α-amylase and amyloglucosidase (E-AMGDF, Megazyme International; Wicklow, Ireland).

### Ruminal infusions and fluid sampling

On d 16 of each experimental period, each animal was fitted with 2 rumen fluid sampling devices consisting of tygon tubing terminating in a plastic mesh pot scrubber weighted with steel nuts. One pot scrubber was placed in the cranial portion of the rumen and the other in the caudal portion with the free ends of tygon tubing exiting the rumen via holes drilled in the cannula plug. Rumen fluid was collected by withdrawing 2.5 mL from both tube ends using a 60 mL luer lok syringe. The sample would then be aliquoted into 2 glass vials and frozen at -21 °C until analysis. A 113 mL polyethylene glycol (PEG) bolus (11.2 g PEG dissolved in 100 mL of water) was administered intraruminally via the rumen cannula at 0500 h on d 17 to 19. Continuous intraruminal infusions of VFA isotope were carried out using Plum A + infusion pumps (Abbott Laboratories; IL, USA). Isotope solutions were infused individually at a rate of 100 mL/h for 6 h with randomization of infusion order across animals. Morning infusions began immediately after PEG bolusing at 0500 h and afternoon infusions began at 1400 h. The following isotopes (Cambridge Isotope Laboratories; Andover, MA) were utilized: Na-2-^13^C-acetate (99% enrichment; solution concentration: 0.052%), Na-2-^13^C-propionate (99% enrichment; solution concentration: 0.052%), Na-2-^13^C-butyrate (99% enrichment; solution concentration: 0.026%), 2-^13^C-valeric acid (99% enrichment; solution concentration: 0.013%), 2-^13^C-isovaleric acid (99% enrichment; solution concentration: 0.0052%), and 2-^13^C-isobutyric acid (99% enrichment; solution concentration: 0.0052%). The isotope infusions were delivered via an IV line attached to a third piece of tygon tubing which was attached to a polyethylene bottle suspended in the rumen. The bottle was drilled with numerous holes in the sides to allow diffusion of the isotope solution and was weighted to maintain a central, upright position in the rumen. Rumen fluid samples were collected at 0430 h, 0445 h, and immediately after the PEG bolus at 0500 h, then hourly until 2300 h on d 17 to 19.

### Determining rumen fluid volume, passage rate, and pH

Polyethylene glycol concentrations of samples taken at 0500 through 1900 h on d 17 of each period were determined following a protocol modification of Smith^49^. Concentrations of PEG were then fitted to an exponential decay curve, the slope of which was taken as the fractional fluid passage rate. An estimation of rumen fluid volume was calculated by dividing the PEG bolus dose by the curve’s y-intercept. Rumen fluid pH was measured on samples taken at 1100 h and 1700 h on d 17–19 using a handheld pH tester (Oakton pH 50 Spear Waterproof Pocket pH Testr, Fisher Scientific; Hampton, NH) immediately following collection.

### Measurement of VFA concentrations

A composite rumen fluid sample was created for each animal using 100 μL of each sample collected during a period day for a total of approximately 2 mL. Volatile fatty acids in each composite sample were derivatized and the concentrations determined by gas chromatography following a procedure adapted from Kristensen^50^. Derivatized samples were analyzed using a Thermo Electron Polaris Q mass spectrometer (Thermo Fisher Scientific; Waltham, MA) in tandem with a Thermo Electron Focus gas chromatograph (Thermo Fisher Scientific; Waltham, MA) with XCalibur software version 1.4 (Thermo Fisher Scientific; Waltham, MA). A Varian FactorFour capillary column VF-170 ms (30 m, 0.25 mm, 0.25 μm) was utilized for VFA differentiation. One microliter of derivatized sample was loaded with the inlet temperature at 225 °C on a split ratio of 80 running a constant flow of helium carrier gas set to 1.2 mL/min. The gas chromatograph was initiated at a temperature of 75 °C, ramped at 5 °C/min to 135 °C, then at 40 °C/min to 225 °C. The mass spectrometer was programmed to run in positive selected ion monitoring mode, collecting 3 consecutive segment *m*/*z* pairs for acetate (43, 46), propionate (57, 62), isobutyrate (71, 78), butyrate (71, 78), isovalerate (85, 94), and valerate (85, 94) in that elution order. The processing method used to integrate the area under the curves for each *m*/*z* used the algorithm of the International Conference on Computer and Information Science.

### Isotope ratio determination and model fitting

Four animals (each receiving the 4 diets in a unique sequence) were randomly selected for isotope ratio determination of their rumen fluid samples. Isotope ratios were determined on individual unpooled samples after acidification with HCl. ^13^C enrichment of the CO_2_ produced from each VFA was assessed using an isotope ratio mass spectrometer (IRMS; Delta V, Thermo Fisher Scientific; Waltham, MA) coupled to a gas chromatograph via a combustion oven (GC-comb-IRMS). Volatile fatty acids were introduced into the GC using a SPME method (SPME autosampler kit for Thermo Tri-Plus; SPME Fiber Assembly, 75um CAR/PDMS, 23ga, Autosampler (Supelco, P/N 57343-U); SPME Liner for TQ, 0.8 mm ID, Straight Through (Supleco, PN 2876601-U), Thermo Scientific). The SPME fiber was exposed to the sample vial’s headspace after heating of the sample at 240 °C for 1 min. The VFA were separated on a Zebron ZB-FFAP column, 30 m × 0.25 mm × 0.25 um (Phenomenex, P/N 7HG-G009-11) operated at 300 °C using helium as a carrier gas at a flow rate of 1.5 mL/min and the data yielded by the analysis were expressed as isotope ratios. Estimates of VFA production, interconversion, and absorption rates were derived by using the isotope ratios, VFA concentrations, and rumen fluid volume and passage rate data to fit a dynamic mechanistic model of these VFA fluxes^41^. The standard 3-pool interchanging model^51^ was updated to reflect a 6-pool system with minor VFA to represent the tracer movements that occurred during continuous infusion of labeled VFA into each of the pools The isotopic enrichment of each pool was described by differential equations and solved using a 4th order Runge Kutta integration algorithm. Volatile fatty acid production and interconversions were estimated by model fitting based on the fluxes and rates of change that were defined for each of the pools^51^ and used as model inputs. Passage of VFA from the rumen via fluid was determined using VFA concentration data and fluid passage measurements. Ruminal absorption of VFA was calculated as the VFA produced or interconverted but not passed with fluid. The FME package of R^52^ was used to fit individual models for each infusion of each of the 4 selected animals.

### Papillae collection and gene expression analysis

Rumen papillae biopsies were collected on d 20 by first inverting a small portion of the ventral sac of the rumen through the cannula and severing 60–80 papillae from the epithelium with cuticle scissors. Phosphate buffered saline (Potassium Phosphate Monobasic 210 mg/L; Sodium Chloride 9000 mg/L; Sodium Phosphate Dibasic, 726 mg/L, Gibco Life Technologies; Grand Island, NY) was used to clean rumen fluid and debris from the papillae before collection. Severed papillae were immediately placed into cryovials containing RNALater (QIAGEN; Valencia, CA) to avoid RNA degradation and were stored at − 80 °C. Isolation of RNA was performed using an RNA Plus Mini Kit (QIAGEN; Valencia, CA) and adequate quality of the RNA was confirmed by concentration (ng/μL) and 260/280 ratio (Supplementary Table S2). Complementary DNA (cDNA) was prepared from the RNA using an Applied Biosystems High-Capacity cDNA Reverse Transcription Kit (Thermo Fisher Scientific; Waltham, MA) and synthesized on a Mastercycler. The genes selected for analysis by Real-Time reverse transcription quantitative PCR (qRTPCR) included those with significant roles in VFA transport, ion transport, VFA metabolism, or epithelial integrity. These 12 genes (Supplementary Table S3) were the monocarboxylate transporters 1, 2, and 4 (MCT1, MCT2, MCT4), sodium-hydrogen exchanger isoforms 1, 2, and 3 (NHE1, NHE2, NHE3), beta-hydroxybutyrate dehydrogenase type 1 (BDH1), 3-hydroxy-3-methylglutaryl-CoA synthase 2 (HMGCS2), heat shock protein 70 (HSP70), serine-threonine protein kinase (AKT1), Claudin-1 (CLDN1), and gap-junction protein alpha 1 (GJA1). Ribosomal protein subunit 9 (RPS9) was utilized as the housekeeper gene to be run with all subsequent genes for comparison. All Real-Time qRTPCR was performed following the method of Lu et al.^53^. Briefly, 1.0 μL of cDNA combined with 9.0 μL of a primer Master Mix was pipetted onto an ABI MicroAmp Fast Optical 96-well reaction plate (Fisher Scientific; Hampton, NH) in triplicate and covered with an optical adhesive cover. The Master Mix was created by combining 0.5 μL of the target gene’s forward primer, 0.5 μL of the target gene’s reverse primer, 5.0 μL of SYBR Fast Green Master Mix (Fisher Scientific; Hampton, NH), and 3.0 μL of molecular-grade water. Plates were run on the Real-Time Fast machine (Applied Biosystems 7500 Real-Time PCR, Thermo Fisher Scientific; Waltham, MA).

### Statistical analysis

Statistical analyses were conducted in R version 3.6.1^52^ using the nlme package^54^. Response variables included DMI (kg/d); fluid volume (L); fluid passage rate (%/h); fluid pH; VFA concentrations (mM); VFA molar proportions (% mol); VFA synthesis, interconversion, and absorption rates (mmol/mmol/h); VFA fluxes (mmol/h); and epithelial gene expression (2^−ΔCt^). Response variables were analyzed using the following linear model:

$$Y_{ijkl} = \mu + \alpha_{i} + \beta_{j} + \alpha \beta_{ij} + c_{k} + d_{l} + e_{ijkl}$$

where *μ* represents the overall mean, *α*_*i*_ is the effect of the *i*th protein source, *β*_*j*_ is the effect of the jth fiber source, *αβ*_*ij*_ is the interaction of protein source *i* and fiber source *j*, *c*_*k*_ is the random effect of animal *k*, *d*_*l*_ is the random effect of period *l*, and *e*_*ijkl*_ is the residual error associated with protein source *i*, fiber source *j*, animal *k*, and period *l*. A number of residual error variance structures, including 1st Order Autoregressive, Unstructured, and Compound Symmetry, were compared for each of the response variables. Model quality was determined based on Akaike information criterion (AIC) and the model possessing the lowest AIC was selected for significance testing. Analysis of variance (ANOVA) was performed on each model and least square means calculated. Significance level was set at *P* < 0.05 and a tendency considered when 0.05 ≤ *P* < 0.10.

## Supplementary Information

Supplementary Tables.

## Data Availability

The data generated and analyzed during the current study are available from the corresponding author on reasonable request.
