# Supplementary material for: Rumen fermentation and epithelial gene expression responses to diet ingredients designed to differ in ruminally degradable protein and fiber supplies
Source: Sci Rep. 2022 Feb 21;12:2933. doi: 10.1038/s41598-022-06890-5 (PMC8861106; doi:10.1038/s41598-022-06890-5)
Supplement: Supplementary file 1 — Supplementary Tables. [file 41598_2022_6890_MOESM1_ESM.docx]

**Supplementary tables**

| **Supplementary Table S1.** LS means for nonsignificant VFA interconversions as differentiated by diet along with *P* values for the effects of protein source, fiber source, and the interaction of protein and fiber source^1,2^ | | | | | | | | |
| --- | --- | --- | --- | --- | --- | --- | --- | --- |
|  | Diet | | | |  | *P* value | | |
| Measurement | SBM-TH | HSBM-TH | SBM-BP | HSBM-BP | SEM | Protein | Fiber | Protein × Fiber |
| Interconversion rate, mmol/mmol/h | |  |  |  |  |  |  |  |
| Acetate to butyrate | 0.304 | 0.232 | 0.308 | 0.122 | 0.11 | 0.23 | 0.61 | 0.59 |
| Acetate to valerate | 0.0706 | 0.0303 | 0.0706 | 0.0741 | 0.019 | 0.29 | 0.20 | 0.20 |
| Acetate to isobutyrate | 0.0280 | 0.0291 | 0.0308 | 0.0522 | 0.013 | 0.29 | 0.23 | 0.34 |
| Propionate to acetate | 0.388 | 0.480 | 0.297 | 0.232 | 0.16 | 0.93 | 0.32 | 0.64 |
| Propionate to isobutyrate | 0.0222 | 0.0298 | 0.0353 | 0.0369 | 0.013 | 0.71 | 0.42 | 0.81 |
| Butyrate to valerate | 0.103 | 0.0231 | 0.0362 | 0.0444 | 0.035 | 0.26 | 0.47 | 0.18 |
| Butyrate to isovalerate | 0.0169 | 0.0182 | 0.0162 | 0.0301 | 0.011 | 0.45 | 0.58 | 0.53 |
| Butyrate to isobutyrate | 0.0106 | 0.0163 | 0.0161 | 0.0330 | 0.0088 | 0.19 | 0.20 | 0.50 |
| Valerate to propionate | 0.293 | 0.325 | 0.390 | 0.357 | 0.12 | 0.99 | 0.57 | 0.77 |
| Valerate to butyrate | 0.0666 | 0.0876 | 0.0656 | 0.0632 | 0.040 | 0.77 | 0.70 | 0.72 |
| Valerate to isobutyrate | 0.0441 | 0.0992 | 0.0779 | 0.0802 | 0.042 | 0.48 | 0.85 | 0.51 |
| Isovalerate to acetate | 0.249 | 0.327 | 0.312 | 0.209 | 0.099 | 0.88 | 0.75 | 0.30 |
| Isovalerate to propionate | 0.313 | 0.204 | 0.225 | 0.222 | 0.083 | 0.51 | 0.67 | 0.53 |
| Isovalerate to butyrate | 0.200 | 0.376 | 0.254 | 0.404 | 0.11 | 0.17 | 0.71 | 0.91 |
| Isovalerate to valerate | 0.0532 | 0.0602 | 0.151 | 0.135 | 0.071 | 0.95 | 0.25 | 0.87 |
| Isovalerate to isobutyrate | 0.0308 | 0.0683 | 0.0440 | 0.0535 | 0.018 | 0.18 | 0.96 | 0.40 |
| Isobutyrate to acetate | 0.198 | 0.207 | 0.176 | 0.276 | 0.11 | 0.64 | 0.84 | 0.69 |
| Isobutyrate to propionate | 0.149 | 0.176 | 0.298 | 0.203 | 0.10 | 0.69 | 0.32 | 0.48 |
| Isobutyrate to valerate | 0.0954 | 0.121 | 0.0694 | 0.134 | 0.069 | 0.48 | 0.92 | 0.76 |
| Isobutyrate to isovalerate | 0.0362 | 0.0486 | 0.0715 | 0.0679 | 0.025 | 0.83 | 0.20 | 0.69 |
| Interconversion flux, mmol/h | |  |  |  |  |  |  |  |
| Acetate to isobutyrate | 0.478 | 0.458 | 0.538 | 1.12 | 0.30 | 0.19 | 0.10 | 0.16 |
| Propionate to acetate | 4.44 | 4.17 | 1.58 | 2.27 | 1.9 | 0.91 | 0.22 | 0.80 |
| Propionate to butyrate | 2.50 | 3.50 | 1.18 | 1.53 | 0.99 | 0.50 | 0.12 | 0.74 |
| Propionate to valerate | 0.809 | 0.811 | 0.349 | 0.682 | 0.34 | 0.58 | 0.34 | 0.59 |
| Propionate to isobutyrate | 0.272 | 0.210 | 0.339 | 0.469 | 0.17 | 0.78 | 0.20 | 0.43 |
| Butyrate to acetate | 1.76 | 1.93 | 1.46 | 2.17 | 0.68 | 0.43 | 0.95 | 0.63 |
| Butyrate to propionate | 1.72 | 1.57 | 2.00 | 1.95 | 0.74 | 0.86 | 0.56 | 0.93 |
| Butyrate to valerate | 0.278 | 0.190 | 0.172 | 0.645 | 0.18 | 0.31 | 0.35 | 0.15 |
| Butyrate to isovalerate | 0.0476 | 0.118 | 0.157 | 0.601 | 0.20 | 0.19 | 0.14 | 0.33 |
| Butyrate to isobutyrate | 0.0471 | 0.128 | 0.115 | 0.515 | 0.20 | 0.22 | 0.25 | 0.41 |
| Valerate to acetate | 0.608 | 0.320 | 0.259 | 0.964 | 0.33 | 0.54 | 0.66 | 0.16 |
| Valerate to propionate | 0.352 | 0.510 | 0.400 | 0.799 | 0.30 | 0.35 | 0.56 | 0.68 |
| Valerate to butyrate | 0.0465 | 0.115 | 0.0532 | 0.1195 | 0.056 | 0.17 | 0.90 | 0.98 |
| Valerate to isobutyrate | 0.0286 | 0.152 | 0.0836 | 0.188 | 0.080 | 0.16 | 0.55 | 0.91 |
| Isovalerate to acetate | 0.111 | 0.211 | 0.424 | 0.251 | 0.15 | 0.81 | 0.26 | 0.38 |
| Isovalerate to propionate | 0.142 | 0.163 | 0.325 | 0.377 | 0.18 | 0.81 | 0.21 | 0.92 |
| Isovalerate to butyrate | 0.0892 | 0.323 | 0.318 | 0.569 | 0.18 | 0.12 | 0.12 | 0.95 |
| Isovalerate to valerate | 0.0233 | 0.0632 | 0.129 | 0.587 | 0.27 | 0.37 | 0.26 | 0.45 |
| Isovalerate to isobutyrate | 0.0174 | 0.0692 | 0.0502 | 0.105 | 0.036 | 0.13 | 0.32 | 0.97 |
| Isobutyrate to acetate | 0.0873 | 0.0891 | 0.111 | 0.334 | 0.11 | 0.34 | 0.26 | 0.35 |
| Isobutyrate to valerate | 0.0856 | 0.104 | 0.0483 | 0.245 | 0.12 | 0.34 | 0.64 | 0.43 |
| ^1^HSBM-TH = heat-treated soybean meal and timothy hay; SBM-TH = soybean meal and timothy hay; HSBM-BP = heat-treated soybean meal and beet pulp; SBM-BP = heat-treated soybean meal and beet pulp.  ^2^mmol/mmol/h = fraction of carbon per hour. | | | | | | | | |

| **Supplementary Table S2.** Quality measurements for RNA extracted from rumen epithelial samples | | | |
| --- | --- | --- | --- |
| Animal ID | Period | 260/280 Ratio | RNA concentration, ng/µL |
| 93 | 1 | 2.06 | 821.1 |
| 219 | 1 | 2.06 | 390.1 |
| 222 | 1 | 2.03 | 115.7 |
| 249 | 1 | 2.05 | 432.9 |
| 302 | 1 | 2.04 | 416.3 |
| 337 | 1 | 2.05 | 877.8 |
| 512 | 1 | 2.06 | 912.7 |
| 529 | 1 | 2.04 | 442.1 |
| 533 | 1 | 2.05 | 104.3 |
| 544 | 1 | 2.04 | 745.0 |
| 93 | 2 | 2.05 | 1092.0 |
| 219 | 2 | 1.94 | 137.3 |
| 222 | 2 | 2.04 | 717.6 |
| 249 | 2 | 2.03 | 921.5 |
| 302 | 2 | 2.03 | 686.2 |
| 337 | 2 | 1.88 | 107.9 |
| 512 | 2 | 2.04 | 1158.8 |
| 529 | 2 | 2.06 | 1478.6 |
| 533 | 2 | 2.03 | 432.0 |
| 544 | 2 | 2.03 | 505.4 |
| 93 | 3 | 1.99 | 750.6 |
| 219 | 3 | 2.05 | 2787.6 |
| 222 | 3 | 1.97 | 1119.1 |
| 302 | 3 | 2.05 | 2149.6 |
| 337 | 3 | 2.06 | 647.9 |
| 512 | 3 | 2.05 | 1650.4 |
| 529 | 3 | 2.06 | 1235.9 |
| 533 | 3 | 2.06 | 2652.5 |
| 544 | 3 | 2.02 | 953.5 |
| 93 | 4 | 2.05 | 2125.0 |
| 219 | 4 | 2.05 | 737.9 |
| 222 | 4 | 2.05 | 1349.9 |
| 302 | 4 | 2.04 | 1347.4 |
| 337 | 4 | 2.04 | 1428.1 |
| 512 | 4 | 2.04 | 1392.1 |
| 529 | 4 | 2.05 | 1836.9 |
| 533 | 4 | 2.05 | 2096.1 |
| 544 | 4 | 2.04 | 1976.3 |

| **Supplementary Table S3**. Primers used in rumen epithelial gene expression analysis | | | |
| --- | --- | --- | --- |
| Primer | Sequence | Accession number | Amplicon size (bp) |
| Beta-hydroxybutyrate dehydrogenase (BDH1) | Fwd: CCCACCACCAGTCTGAGCAT  Rev: CCCACTACTCTGCACCCCAA | NM_001034600.1 | 101 |
| Claudin-1 (CLDN1) | Fwd: CAGTGCAAAGTCTTCGACTCC  Rev: GTCGTCTTCCATGCACTTCA | BT021861.1 | 147 |
| Gap-junction protein alpha 1 (GJA1) | Fwd: ATGAGCAGTCTGCCTTTCGT  Rev: AGCCAGGTACAGGAGTGTGG | NM_174068.2 | 143 |
| Heat shock protein 70 (HSP70) | Fwd: AGCTGGAGCAGGTGTGTAAC  Rev: AGCTTGCATAGCTGATGGCT | U09861.1 | 239 |
| 3-Hydroxy-3-methylglutaryl-CoA synthase 2 (HMGCS2) | Fwd: TTACGGGCCCTGGACAAAT  Rev: CACATCATCGAGAGTGAAAGG | NM_001045883.1 | 100 |
| Monocarboxylic acid transporter 1 (MCT1) | Fwd: GTCATTGGAGGTCTTGGGCT  Rev: GGTAGAGAGGAACACAGGGC | NM_001037319.1 | 129 |
| Monocarboxylic acid transporter 2 (MCT2) | Fwd: TGGTCTCGGCCTCTTACAGT  Rev: GCCATTCGCTACAGGTCGTT | NM_001076336.2 | 146 |
| Monocarboxylic acid transporter 4 (MCT4) | Fwd: GTGACACAGCCTGGATCTCC  Rev: AGAAGGACGCAGACACCATG | NM_001109980.1 | 150 |
| Serine-threonine protein kinase 1 (AKT1) | Fwd: CTGCACAAGCGAGGTGAGTA  Rev: GAAGTTGTTGAGGGGCGACT | NM_173986.2 | 132 |
| Sodium-hydrogen exchanger 1 (NHE1) | Fwd: GTCCCACACGACCATCAAGT  Rev: AGGGTGCTGATGACAAACGT | NM_174833.2 | 132 |
| Sodium-hydrogen exchanger 2 (NHE2) | Fwd: CGAGCAGCTCTACATCCTGG  Rev: ATGCCAGCAAACACGTCAAC | XM_604493.6 | 129 |
| Sodium-hydrogen exchanger 3 (NHE3) | Fwd: CCTCATGAGAAGGTCGGCTC  Rev: GAACGGATGAAAGCCAGGGA | NM_001192154.1 | 129 |
| Ribosomal protein subunit 9 (RPS9) | Fwd: GTGAGGTCTGGAGGGTCAAA  Rev: GGGCATTACCTTCGAACAGA | NM_001101152.2 | 108 |
